# Supplementary material for: Role of Running-Activated Neural Stem Cells in the Anatomical and Functional Recovery after Traumatic Brain Injury in p21 Knock-Out Mice
Source: Int J Mol Sci. 2023 Feb 2;24(3):2911. doi: 10.3390/ijms24032911 (PMC9918280; doi:10.3390/ijms24032911)
Supplement: Supplementary file 1 [file ijms-24-02911-s001.zip › ijms-2117820-supplementary.pdf]

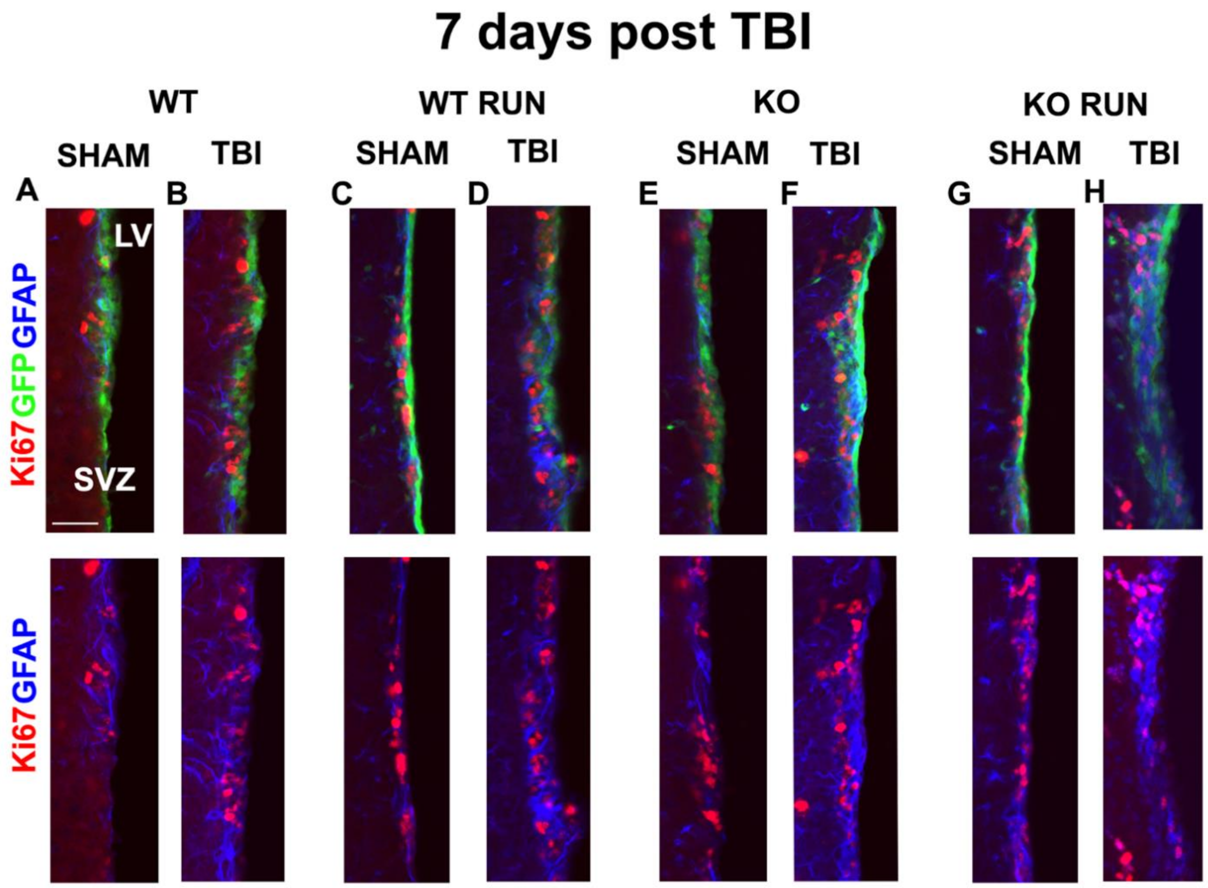

Supplemental figure S1. Confocal representative images showing the increased NSCs recruitment and proliferation in TBI-subjected groups (1 B, D, F, H) respect to their SHAM counterparts (1 A, C, E, G) at 7-days post TBI. Magnification 20x. Bar = 50 μm. LV =lateral ventricle, SVZ = subventricular zone.

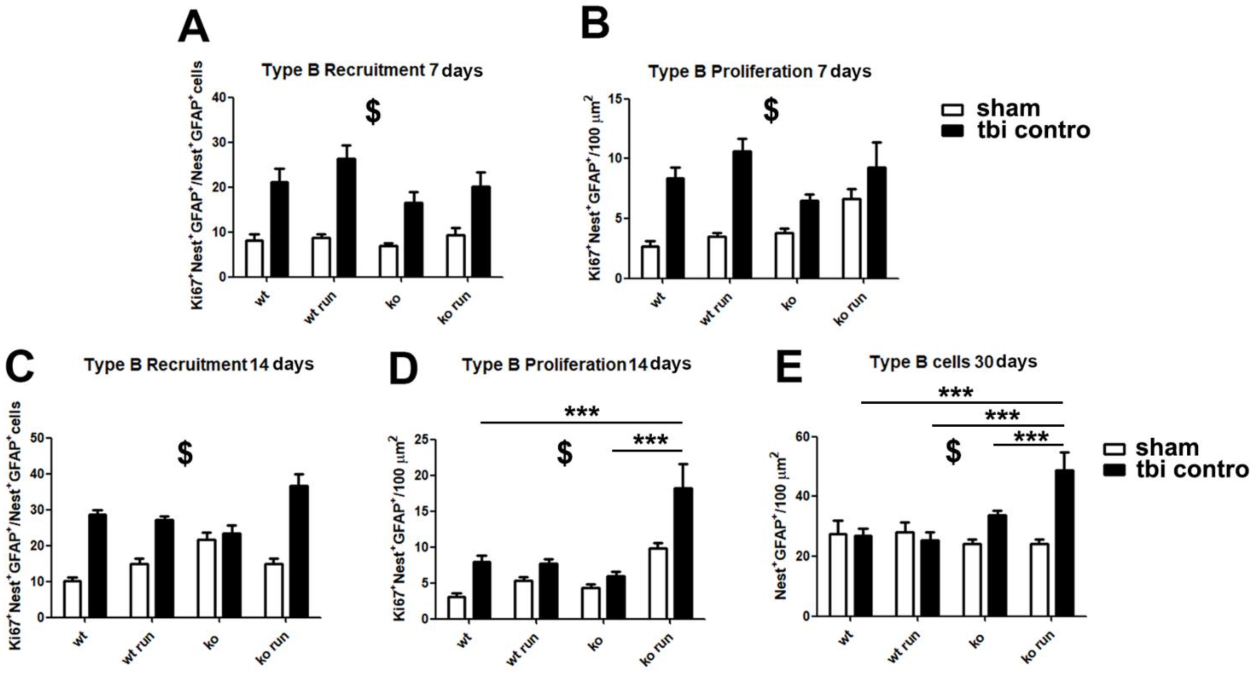

Supplemental figure S2. **(A, B)** Graphs showing the increase at 7-days post TBI of Type B recruitment (lesion effect: $F_{(1,57)} = 75.38$, p <0.001A, $) and proliferation (lesion effect: $F_{(1,60)} = 44.6$, p <0.001, Suppl. Fig. 1 B, $) in contralateral SVZ of the mice underwent to the TBI in comparison to their SHAM. (**C, D**) Histograms indicating the increase at 14-days post TBI of Type B recruitment (lesion effect: $F_{(1,56)} = 113$, p <0.001, C, $) and proliferation (lesion effect: $F_{(1,56)} = 20.8$, p <0.001, D, $) in contralateral SVZ of the mice underwent to the TBI in comparison to their SHAM. In the panel D, it is possible to detect a TBI-dependent expansion of Type B cells in the KO RUN TBI respect to the other groups in term of enhancement of Type B proliferation ($Ki67^+$ GFP^+ $GFAP^+$ cells: genotype x run interaction: $F_{(1,60)} = 17,42$, $p < 0.001$, followed by post-test LSD, KO RUN TBI vs WT TBI and KO TBI, $p < 0.001$). (**E**) Graph showing the enhanced Type B pool size in the contralateral SVZ of TBI groups compared to their SHAM littermates (contra-lateral: lesion effect: $F_{(1,44)} = 10.25$, p = 0.002, $). Asterisks (***) indicate the significant increase of Type B cell density in the KO RUN TBI mice compared the other TBI groups (GFP^+ $GFAP^+$ cells: cells: genotype x run interaction: $F_{(1,44)} =$ 5,53, p = 0.022, followed by post-test LSD, KO RUN TBI vs WT TBI, WT RUN TBI and KO TBI, $p < 0.001$). Statistical significance of LSD post-hoc analysis: *** $p < 0.001$. Statistical significance

of lesion main effect between SHAM and TBI groups: $ p < 0.001. Multifactorial analysis with three independent variables: genotype, treatment and running, followed by by Fisher's LSD post hoc tests.

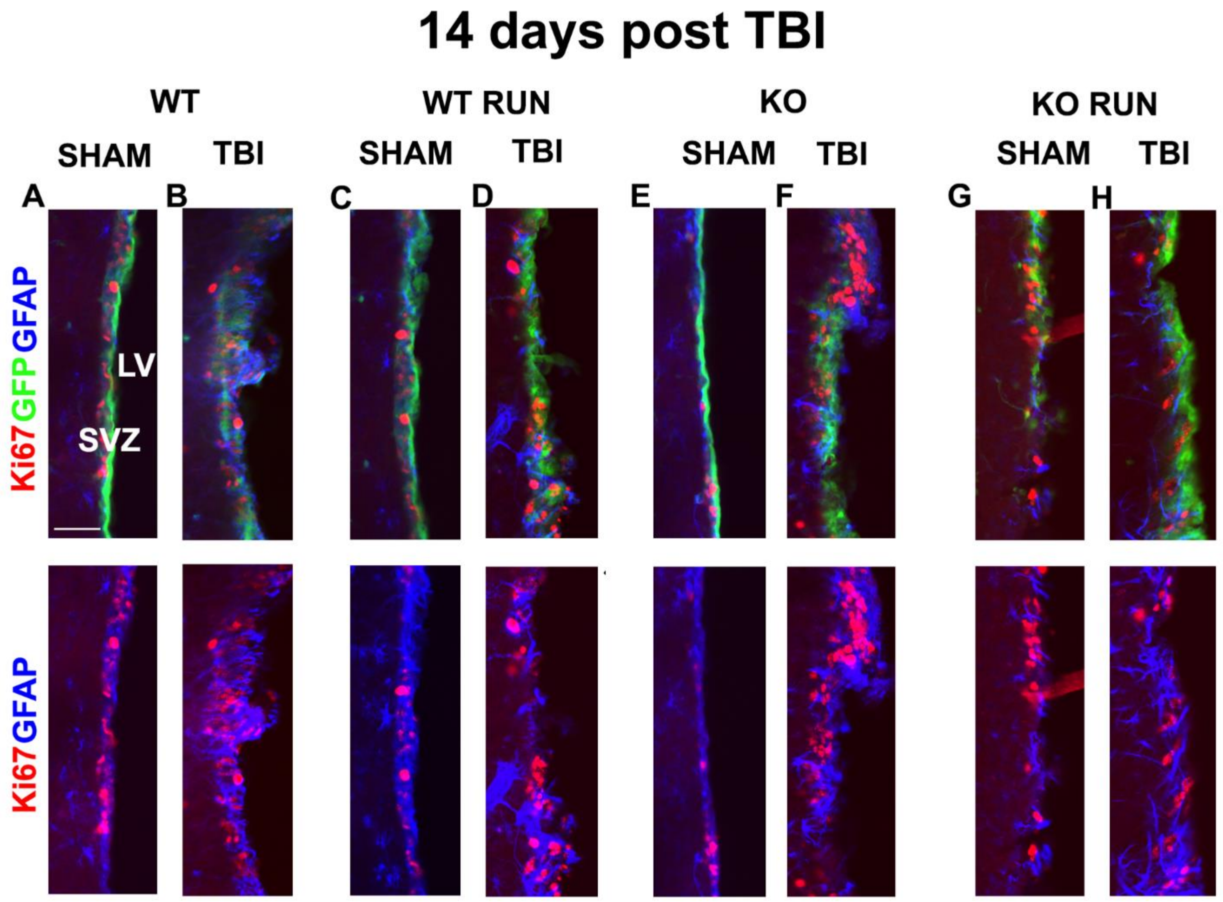

Supplemental figure S3. Confocal images representing the significant enhancement of NSCs recruitment and proliferation in the SVZ of TBI groups (3 B, D, F, H) respect to the SHAM littermates (3 A, C, E, G) at 14-days post TBI. Magnification 20x. Bar = 50 µm. LV =lateral ventricle, SVZ = subventricular zone.

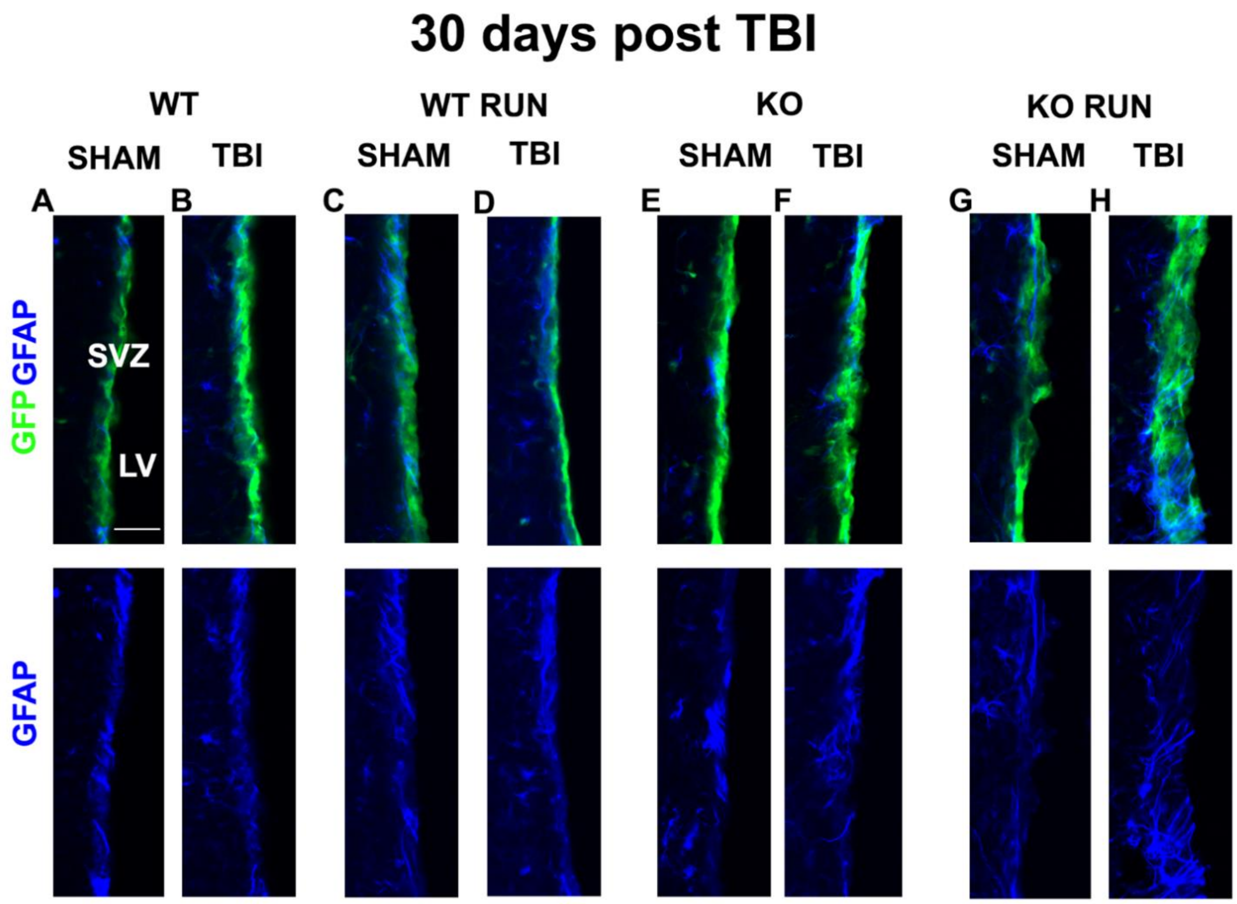

Supplemental figure S4. Representative images illustrating the expansion of Type B cell pool size in the KO TBI (F) and KO RUN TBI (H) mice comparison to their SHAM counterparts (KO SHAM, E and KO RUN SHAM, G), 30-days after TBI. Magnification 20x. Bar = 50 µm. LV =lateral ventricle, SVZ = subventricular zone.

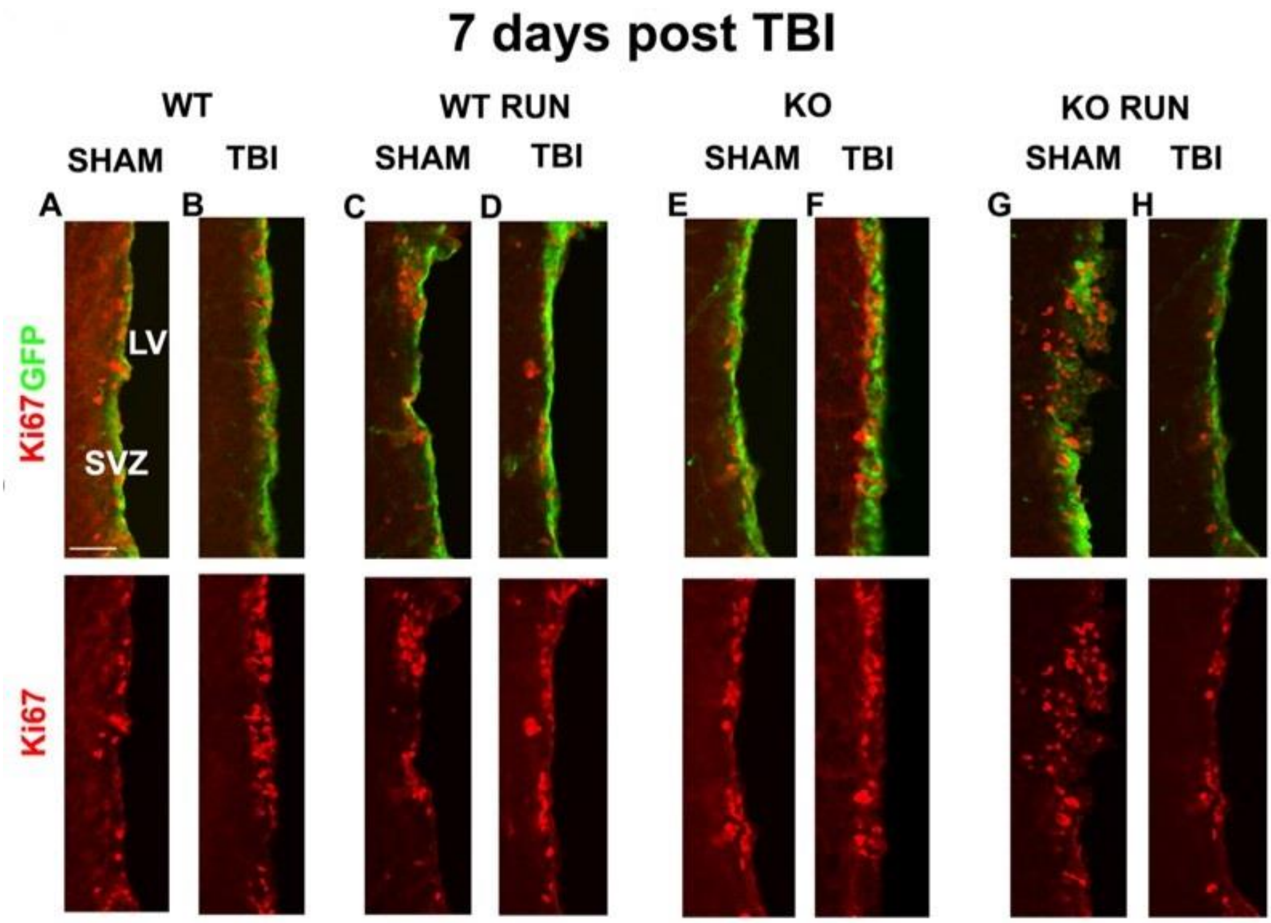

Supplemental figure S5. Confocal micrographs showing at 7-days post TBI an increasing NSPCs proliferation in the WT TBI mice (B) and in KO TBI mice (F) respect to their SHAM littermates, WT SHAM (A) and KO SHAM (E). G and H show the decreased NSPCs proliferation in KO RUN TBI (H) respect to KO RUN SHAM (G) mice. Magnification 20x. Bar = 50 µm. LV =lateral ventricle, SVZ = subventricular zone.

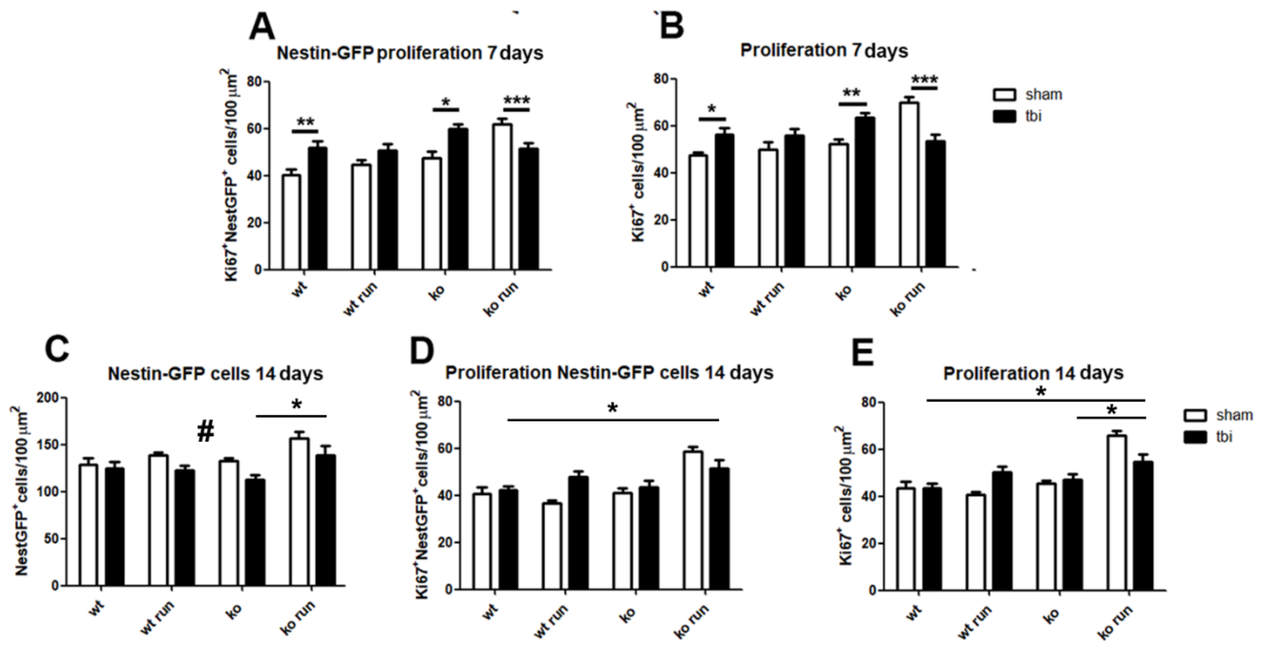

Supplemental figure S6. (**A, B**) Graphs showing at 7 days after TBI the increased Nestin-GFP (contralateral: genotype x run x lesion interaction: $F_{(1,94)} = 5.35$, $p < 0.001$, followed by post-test LSD, WT TBI vs WT SHAM $p = 0.0045$, KO TBI vs KO SHAM $p = 0.013$, A) and total proliferation (genotype x run x lesion interaction, $F_{(1,105)} = 12.29$, $p < 0.001$, followed by post-test LSD, WT TBI vs WT SHAM, $p = 0.019$, and KO TBI vs KO SHAM $p = 0.0013$, B) in the contralateral SVZ of WT TBI and KO TBI mice respect to their SHAM group, and the concomitant decrease of these two parameters in the KO RUN TBI respect to the KO RUN SHAM. (**C**) Histogram showing the decrease density of Nestin GFP$^+$ cells in TBI animals in comparison with their respective SHAM groups 14 day after TBI (lesion effect: $F_{(1,93)} = 11.33$, $p = 0.0011$, #). Asterisk (*) indicates the significantly increase of Nestin GFP$^+$ cell in the contralateral SVZ of KO RUN TBI respect to the KO TBI mice. (**D, E**) The graphs show that 14-day after TBI there is an increased density of proliferating Nestin GFP$^+$ cells in the contralateral SVZ of KO RUN TBI respect to the WT TBI (Ki67$^+$ NestinGFP$^+$: genotype x run interaction: $F_{(1,94)} = 11.56$, $p < 0.001$, followed by LSD post-test, KO RUN TBI vs WT TBI, $p = 0.024$, D) and an enhancement in of total proliferation in the KO RUN TBI respect to the WT TBI and KO TBI groups (Ki67$^+$: genotype x run interaction: $F_{(1,92)} = 12.2$, $p < 0.001$, followed

by LSD post-test , KO RUN TBI vs WT TBI, $p = 0.0048$, vs KO TBI, $p = 0.006$, E). Statistical significance of LSD post-hoc analysis: * $p < 0.05$, ** $p < 0.01$ and *** $p < 0.001$. Statistical significance of lesion main effect between SHAM and TBI groups: # $p < 0.01$. Multifactorial analysis with three independent variables: genotype, treatment and running, followed by by Fisher's LSD post hoc tests.

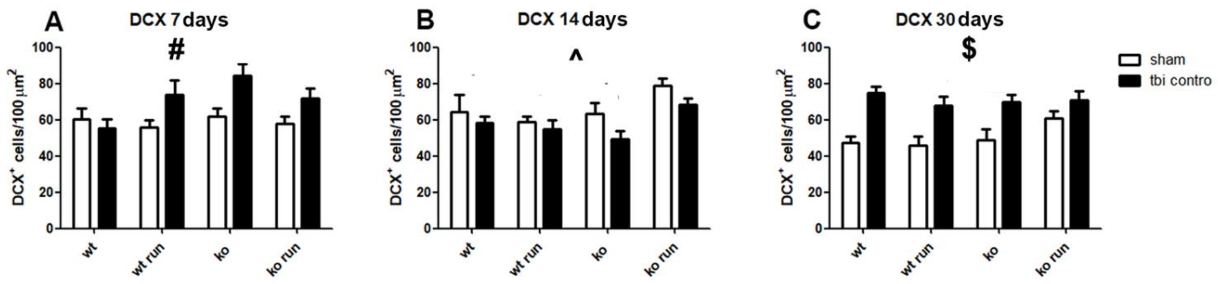

Supplemental figure S7. (**A**) Graph showing the increased number of Dcx^{+} cells in the contralateral SVZ of WT RUN TBI, KO TBI and KO RUN TBI mice, in comparison to their SHAM counterparts 7-day post TBI. (lesion effect: $F_{(1,65)} = 8.68$, $p = 0.004$, #). (**B**) Histogram indicating the decreased number of Dcx+ cells in the contralateral SVZ of TBI groups respect to the SHAM mice, 14 days after TBI (lesion effect, $F_{(1,61)} = 4.97$, $p <0.029$, ^). (**C**) Histogram indicating the enhanced density of Dcx+ cells in the contralateral SVZ of TBI groups respect to the SHAM mice, 30 days after TBI lesion effect: $F_{(1,54)} = 32.8$, $p <0.001$ $). Statistical significance of lesion main effect between SHAM and TBI groups: $ $p < 0.001$, # $p < 0.01$, ^ $p < 0.05$. Multifactorial analysis with three independent variables: genotype, treatment and running, followed by by Fisher's LSD post hoc tests.

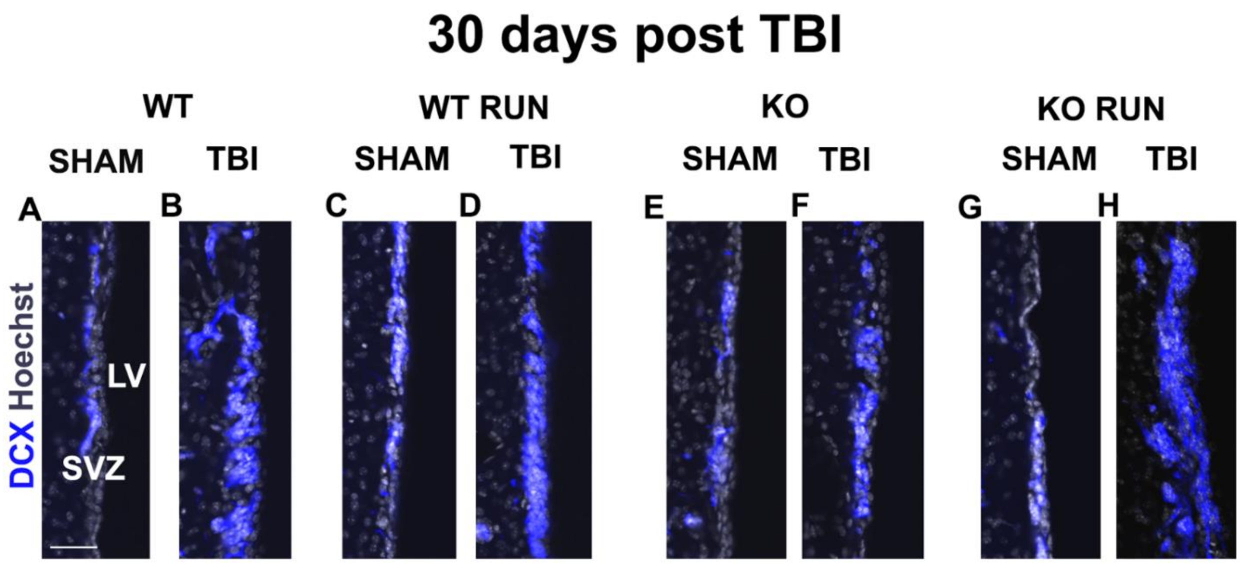

Supplemental figure S8. Representative images illustrating at 30-days post TBI the significant enhancement of DCX$^+$ neuroblasts pool size in the TBI groups (B, D, F, H) respect to their SHAM counterparts (A, C, E, G). Moreover, the images show the large increase of DCX$^+$ cells in the KO RUN TBI mice (H) respect to the other TBI groups (B, D, F). Magnification 20x. Bar = 50 µm. LV =lateral ventricle, SVZ = subventricular zone.

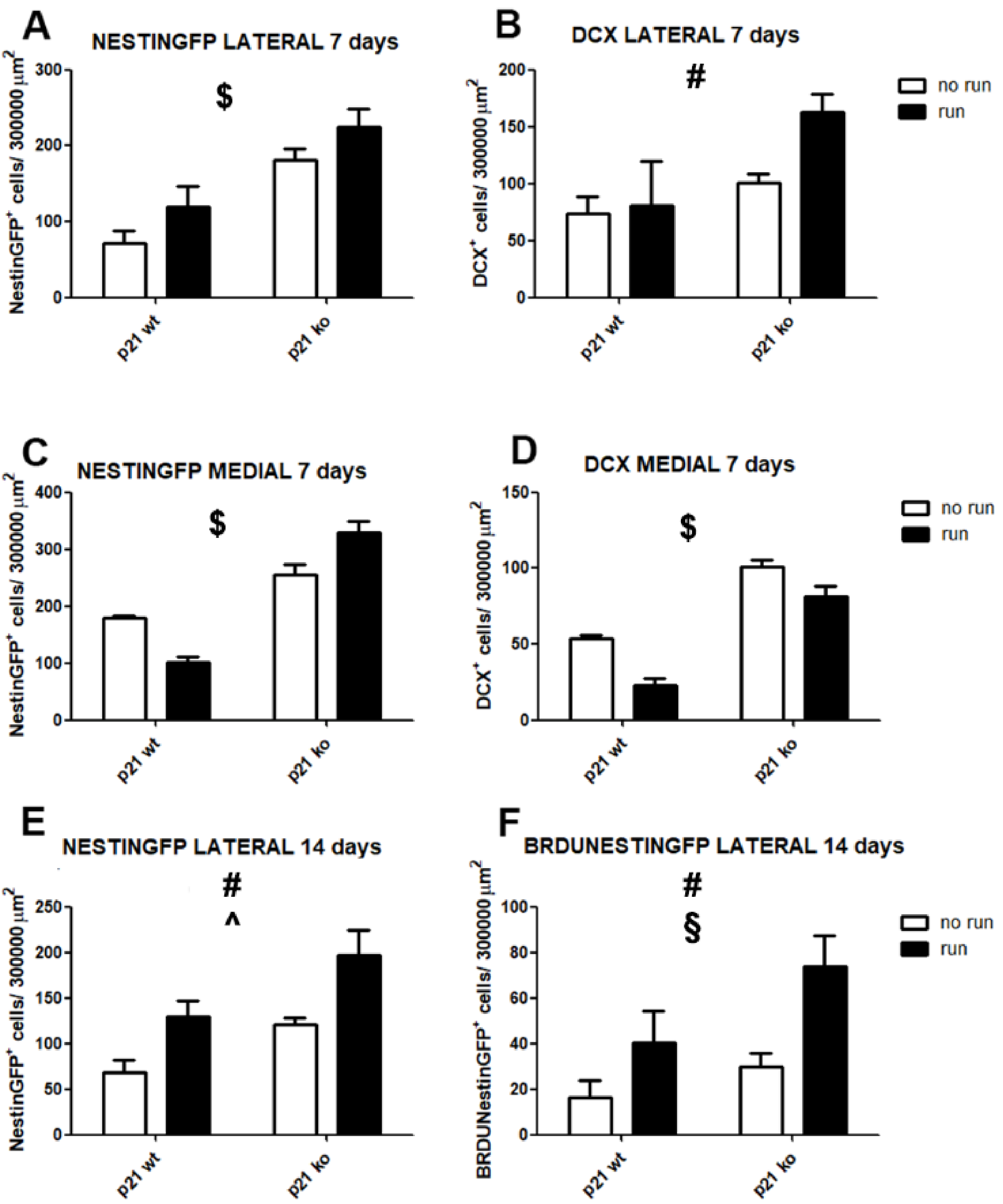

Supplemental figure S9. (**A-B**) The graphs show that after 7 days from TBI there is an increase of NestinGFP$^+$ (genotype effect: $F_{(1, 24)} = 20.32$, $p < 0.001$, A, $) and DCX$^+$ cells (genotype effect: F (1, 24) = 6.12, p = 0.002, #) in the lateral side of the lesion of KO TBI and KO RUN TBI respect to their WT counterpart. (**C-D**) The graphs indicate that after 7 days from TBI there is an increase of NestinGFP$^+$ (genotype effect: F (1, 21) = 24.48, p <0.001, C, $) and DCX$^+$ cells (genotype effect: F

(1, 24) = 108, $p < 0.001$, D, $) in the medial side of the lesion of KO TBI and KO RUN TBI respect to their WT counterpart. **(E-F**) The histograms indicate a genotype and run effect 14 days after TBI in the NestinGFP$^+$ (genotype effect: $F_{(1,20)} = 14.04$, $p = 0.0013$, #; run effect: F (1.20) = 10.7, p = 0.0038, E, ^) and BrdU$^+$/NestinGFP$^+$ cells localized in the lateral cortical regions of the lesion (genotype effect: $F_{(1,20)} = 9.65$, $p = 0.0056$, #; run effect: $F_{(1.20)} = 4.64$, $p = 0.043$, §, 4 F). Statistical significance of genotype main effect between WT and KO groups: $ $p < 0.001$, # $p < 0.01$. Statistical significance of running main effect between WT and KO groups: ^ $p < 0.01$, § $p < 0.05$. Multifactorial analysis with three independent variables: genotype, treatment and running, followed by by Fisher's LSD post hoc tests.
